# Are treatment outcomes in gastric cancer associated with either hospital volume or surgeon volume?

**DOI:** 10.1002/ags3.12031

**Published:** 2017-08-31

**Authors:** Yosuke Mukai, Yukinori Kurokawa, Shuji Takiguchi, Masaki Mori, Yuichiro Doki

**Affiliations:** ^1^ Department of Gastroenterological Surgery Osaka University Graduate School of Medicine Osaka Japan

**Keywords:** gastric cancer, heterogeneity, hospital volume, mortality, surgeon volume

## Abstract

Surgical resection is the only curative treatment for gastric cancer. Postoperative outcomes may be affected by the average or total number of surgeries carried out at an institution (hospital volume) or by a surgeon (surgeon volume). Among seven large‐scale studies that each enrolled over 10 000 patients who underwent gastrectomy, six showed that higher hospital volume contributed to a lower mortality rate after gastrectomy. Surgeon volume was also reported by three of four studies that each included over 1000 patients to be a significant factor contributing to heterogeneity in mortality rates after gastrectomy. In contrast, most studies showed no relationship between hospital volume and postoperative morbidity. A significant long‐term relationship was demonstrated in four of nine studies that each included more than 1000 patients, but the other five studies showed negative results. A recent correlative study of randomized phase III trials for gastric cancer surgeries showed a significant relationship between hospital volume and postoperative morbidity in one trial but not in another trial. There was no correlation between overall survival and either hospital or surgeon volume. In addition, another correlative study of a phase III trial of randomized chemotherapy for unresectable or recurrent gastric cancer found that there was no correlation between hospital volume and overall survival, although there was a large degree of heterogeneity in median overall survival among participating institutions.

## 1 INTRODUCTION

Gastric cancer is the second leading cause of cancer death worldwide,^1^ and surgical resection is the only curative treatment. However, recurrence can be observed even after curative resection for gastric cancer.^2^ As previous studies have shown that the recurrence rate could be significantly affected by the occurrence of postoperative morbidities,^3^, ^4^ we should examine the significant factors associated with short‐term outcomes including postoperative mortality and morbidity. The average and total number of surgeries carried out at an institution (hospital volume) and by an individual surgeon (surgeon volume) are considered factors that affect outcomes after surgery. Several large population‐based studies and systematic literature reviews have shown a close relationship between hospital volume and postoperative mortality in various types of cancer after surgical resection.^5^, ^6^, ^7^, ^8^, ^9^ Patients who underwent surgery at high‐volume hospitals had lower rates of postoperative mortality than those at low‐volume hospitals. For gastric cancer surgery, operative mortality rates were reported to be 13.0% at very‐low‐volume hospitals and 8.7% at very‐high‐volume hospitals in the USA,^5^ compared to 1.1% at low‐volume hospitals and 0.4% at high‐volume or very‐high‐volume hospitals in Japan.^10^ However, fewer studies have evaluated heterogeneity in outcomes after gastrectomy than after other types of surgery. Furthermore, no studies thus far have investigated the correlation between hospital volume and outcomes after chemotherapy for gastric cancer.

Here we review the relationship between postoperative outcomes and both hospital and surgeon volumes in patients with resectable gastric cancer. In addition, we introduce two correlative studies of Japanese randomized phase III trials investigating inter‐institutional heterogeneity in outcomes after gastrectomy for resectable gastric cancer or after chemotherapy for unresectable or recurrent gastric cancer.

## 2 METHODS

### 2.1 Search methods for identification of studies

**Figure 1:**
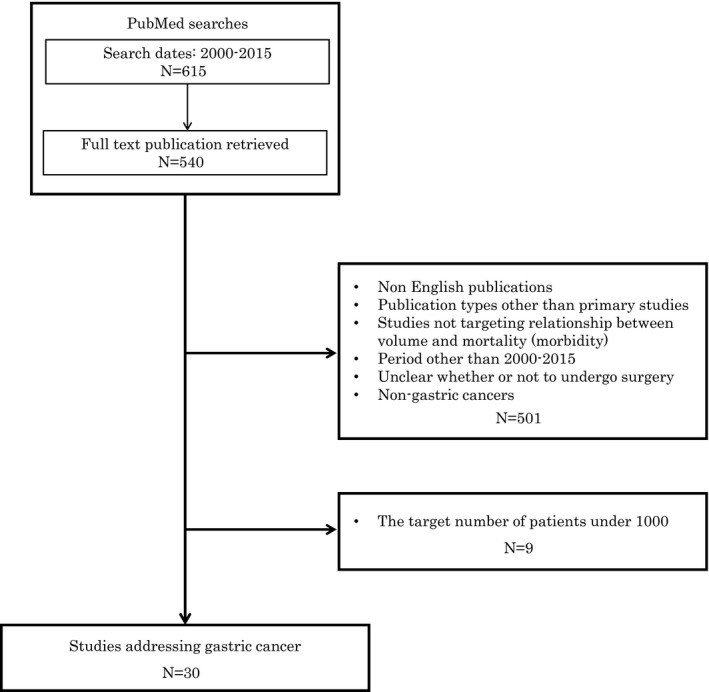
Selection of references in the present study

We conducted a comprehensive search to identify all relevant primary studies addressing the impact of hospital or surgeon volume on patient outcomes after treatment for gastric cancer between 2000 and 2015. We searched the literature using PubMed, with the following search terms: “([volume AND [outcome OR mortality OR morbidity] AND [gastric cancer OR stomach cancer OR gastrectomy]]) AND (“2000”[Date—Publication]:“2015”[Date—Publication])”. We included studies that evaluated short‐term mortality, morbidity, or long‐term survival in gastric cancer patients who underwent surgical intervention. We excluded non‐English publications, narrative reviews, editorials, letters, and case reports, as well as studies with fewer than 1000 gastric cancer patients. Of the 615 studies initially identified in the PubMed search, 30 met the above criteria and were analyzed (Figure 1). Classification of low‐ and high‐volume groups were defined using the cut‐off numbers in the original articles.

## 3 RESULTS

### 3.1 Short‐term outcomes after gastrectomy

Since the 2000s, a total of 23 studies that each included more than 1000 patients have evaluated the relationship between hospital volume and mortality after gastrectomy (Table 1). As a whole, a higher hospital volume was significantly associated with a lower postoperative mortality. Among seven large‐scale studies with over 10 000 patients each, six showed that a higher hospital volume contributed to a significantly lower mortality rate after gastrectomy.^5^, ^11^, ^12^, ^13^, ^14^, ^15^ Birkmeyer et al.^5^ reported that the non‐elective admission rate was higher in very‐low‐volume hospitals than in very‐high‐volume hospitals (51.4% vs 29.7%), and that this discrepancy was possibly associated with a difference in mortality rates (13.0% vs 8.7%). A significant correlation between hospital volume and postoperative mortality was also observed in six studies in the USA 16, 17, 18, 19, 20, 21 and three studies in Europe.^22^, ^23^, ^24^ Among eight other large‐scale studies, only one could not show a significant relationship between higher hospital volume and lower hospital mortality, but the absolute difference in mortality between low‐ and high‐volume hospitals was greater than 1% (8.7% vs 6.9%).^25^

**Table 1:** Studies with more than 1000 patients evaluating the relationship between hospital volume and mortality after gastrectomy

Reference (year)	Country	No. patients	No. hospitals	Hospital volume group (No. gastrectomies per year)	Mortality rates (risk ratio)	*P* value
Damhuis et al. (2002)^41^	Netherlands	1978	22	Low (<7) vs high (>10)	8.0% vs 6.8%	.21
Hannan et al. (2002)^16^	USA	3711	207	Lowest (≤15 per 4 y) vs highest (≥63 per 4 y)	11.16% vs 2.85% (OR, 7.10)	<.0001
Birkmeyer et al. (2002)^5^	USA	31 944	3423	Very high (mean >21) vs very low (mean <5)	8.7% vs 13.0% (OR, 0.72; 95% CI, 0.63‐0.83)	<.001
Finlayson et al. (2003)^25^	USA	16 081	911	High (>17) vs low (<9)	6.9% vs 8.7%	NS
Wainess et al. (2003)^11^	USA	23 690	Unknown	Low (≤4) vs high (≥9)	8.3% vs 6.5% (OR, 1.1; 95% CI, 1.0‐1.3)	<.001
Callahan et al. (2003)^17^	USA	6434	213	Low (≤27 per 4 y) vs high (≥141 per 4 y)	11.3% vs 3.7% (OR, 2.34, 95% CI, 1.40‐3.90)	<.0001
Lin et al. (2006)^12^	Taiwan	11 348	174	Highest vs lowest	1.35% vs 5.35% (OR, 0.28; 95% CI, 0.15‐0.53)	<.05
Smith et al. (2007)^18^	USA	1864	214	High (>15) vs low (<3)	No comorbidity: 0.8% vs 4.1% Comorbidity: 1.7% vs 6.4% (OR, 0.22; 95% CI, 0.05‐0.89)	.04
Birkmeyer et al. (2006)^19^	USA	9403	2934	Lowest vs highest	10.1% vs 7.3% (OR, 1.43; 95% CI, 1.11‐1.83)	<.05
Reid‐Lombardo et al. (2007)^20^	USA	3277	691	Community (mean 2.9) vs teaching/research (mean 7.6)	9.9% vs 5.5%	<.01
Smith et al. (2007)^13^	USA	13 354	Unknown	Lowest (≤4) vs highest (≥11)	6.8% vs 4.9% (OR, 1.5; 95% CI, 1.2‐1.8)	<.001
Pal et al. (2008)^42^	England	8183	155	Low (≤68 per 6 y) vs high (≥69 per 6 y)	6.0% vs 6.2% (OR, 0.97; 95% CI, 0.80‐1.19)	.77
Reavis et al. (2009)^26^	USA	2169	121	Low (≤5) vs high (≥13)	4.4% vs 2.4%	.06
Bare et al. (2009)^43^	Spain	3241	144	Low (<18) vs high (>35)	7.9% vs 11.6% (OR, 1.245; 95% CI, 0.892‐1.736)	.242
Skipworth et al. (2010)^44^	Scotland	4589	23	Lowest (≤3) vs highest (≥10)	8.9% vs 8.6%	NS
Learn & Bach (2010)^14^	USA	19 338	Unknown	High (>9) vs low (≤4)	Absolute difference, 2.8% (OR, 0.99; 95% CI, 0.98‐0.99)	<.001
Anderson et al. (2011)^22^, ^a^	England	2758	Unknown	High (>30) vs low (≤10)	Unknown (HR 0.385)	<.001
Kuwabara et al. (2011)^15^	Japan	17 761	258	High vs low	Unknown (OR, 0.997; 95% CI, 0.994‐0.999)	<.05
Ghaferi et al. (2011)^21^	USA	37 865^b^	Unknown	Very low (mean <2) vs very high (mean >11)	17.7% vs 7.5% (OR, 2.67; 95% CI, 2.24‐3.18)	<.05
Coupland et al. (2013)^23^, ^a^	England	7786	144	Highest (≥80) vs lowest (<20)	4.1% vs 7.3% (HR, 0.52; 95% CI, 0.39‐0.70)	<.0001
Dikken et al. (2013)^24^	Netherlands, Sweden, Denmark, England	9010	Unknown	High (≥21) vs low (≤10)	4.4% vs 6.7% (OR, 0.64; 95% CI, 0.41‐0.99)	.025
Smith et al. (2014)^38^	Australia	1621	84	Low (≤6) vs high (>6)	5.1% vs 3.8% (OR, 1.37; 95% CI, 0.80‐2.33)	.25
Murata et al. (2015)^27^	Japan	5941	741	High (≥40 per 3 y) vs low (<40 per 3 y)	0.3% vs 0.5% (OR, 0.53; 95% CI, 0.20‐1.41)	.200

a These studies included esophageal cancer patients.

b This number included patients who underwent esophagectomy or pancreatectomy.

CI, confidence interval; HR, hazard ratio; NS, not significant; OR, odds ratio.

In contrast, there is less evidence regarding the relationship between hospital volume and postoperative morbidity. To the best of our knowledge, most studies showed no relationship between hospital volume and postoperative morbidity.^18^, ^20^, ^21^, ^26^, ^27^, ^28^ Morbidity rate is generally affected by patient characteristics such as comorbidities, acuity of presentation, and age.^29^ High‐volume hospitals have a great deal of experience treating postoperative morbidities without failure to rescue.^18^, ^21^, ^30^ Thus, differences in mortality rates might be as a result of differences in clinical abilities to treat postoperative complications. Recently, a Japanese study evaluated the relationship between hospital volume and short‐term outcomes in laparoscopic gastrectomy, but there was no significant difference between hospital volume and either laparoscopy‐related complications (odds ratio [OR], 0.96; 95% confidence interval [CI], 0.79–1.16; *P*=.684) or mortality (OR, 0.53; 95% CI, 0.20–1.41; *P*=.208).^27^ Thus, hospital volume may not contribute to postoperative morbidity in either open or laparoscopic gastrectomy.

Surgeon volume may contribute to heterogeneity in postoperative outcomes. Birkmeyer et al.^31^ reported that the majority of hospital volume effect was largely as a result of surgeon volume, and surgeon volume was positively related to postoperative mortality in the USA for all eight procedures they investigated. Unfortunately, their study did not include patients with gastric cancer. In terms of gastrectomy, three of four studies that each included more than 1000 patients showed a relationship between surgeon volume and mortality after gastrectomy (Table 2). Xirasagar et al.^32^ reported that the adjusted hazard ratio (HR) for death within 6 months was 1.3 (*P*<.01) for low‐volume surgeons (6‐month mortality, 23.0%) relative to very high‐volume surgeons (6‐month mortality, 16.7%). Their study also showed that increasing surgeon age, which is correlated with the total number of surgeries carried out, was a significant prognostic factor. Yu et al.^33^ reported that there was no significant association between surgeon volume and short‐term mortality, but a significant difference between specialized and general surgeons was found in the 5‐year survival rate (63.9% vs 59.7%, *P*=.038).

**Table 2:** Studies with more than 1000 patients evaluating the relationship between surgeon volume and mortality after gastrectomy

Reference (year)	Country	No. patients	No. surgeons	Surgeon volume group (No. gastrectomies per year)	Mortality rate (risk ratio)	*P* value
Hannan et al. (2002)^16^	USA	3711	1114	Lowest (<2 per 4 y) vs highest (≥12 per 4 y)	8.83% vs 2.76% (OR, 5.73)	<.0001
Callahan et al. (2003)^17^	USA	6434	1387	Low (≤4 per 4 y) vs high (≥21 per 4 y)	12.3% vs 3.2% (OR, 2.99; 95% CI, 1.79‐4.99)	<.0001
Yu et al. (2005)^33^	Korea	1877	Unknown	General vs specialized^a^	1.6‐2.0% vs 0.9‐1.1%^b^	NS
Xirasagar et al. (2008)^32^	Taiwan	6909	657	Low (≤13 per 3 y) vs very high (≥73 per 3 y)	23.0% vs 16.7%^c^ (HR, 1.3)	<.01

a Surgeon volume was classified into two groups (general or specialized) according to both the number of gastrectomies per year and the consecutive years of practice.

b Describes mortality rates when specialized surgeons had at least four consecutive years of surgical practice.

c Describes 6‐month mortality and hazard ratio.

CI, confidence interval; HR, hazard ratio; NS, not significant; OR, odds ratio.

### 3.2 Long‐term outcomes after gastrectomy

As described above, most studies have focused on the relationship between hospital volume and short‐term outcomes in patients undergoing gastrectomy. Since the 2000s, nine studies that each included at least 1000 patients have focused on long‐term survival after gastrectomy (Table 3), and four of these found a significant relationship between survival and hospital volume.^23^, ^34^, ^35^, ^36^ In a study from the USA showing a positive relationship between hospital volume and long‐term outcomes (HR, 0.82; 95% CI, 0.73–0.92; *P*<.001), differences in outcomes could not be explained by discrepancies in patient characteristics or operative mortality, suggesting that hospital volume was an independent predictor of long‐term survival after gastrectomy.^35^ In contrast, a Dutch study showed that high hospital volume was associated with long‐term survival after esophagectomy, but not after gastrectomy.^37^ A Japanese study showed that in patients with localized or regionally metastasized gastric cancer, 5‐year survival between 1975 and 1979 was worse when resection was carried out at very low‐volume hospitals compared to high‐volume hospitals.^34^ This correlation, however, decreased in later time periods and disappeared in the period between 1990 and 1994, except for very low‐volume hospitals. The authors commented that improvements in medical technology and cancer care were widespread among various types of hospitals in Japan, so the influence of hospital volume on long‐term survival might have diminished for common malignancies such as gastric cancer. However, patients who were treated at very low‐volume hospitals remained at significantly higher risk of death than those treated at high‐volume hospitals.

**Table 3:** Studies with more than 1000 patients evaluating the relationship between hospital volume and long‐term survival after gastrectomy

Reference (year)	Country	No. patients	No. hospitals	Hospital volume group (No. gastrectomies per year)	Long‐term survival rate (risk ratio)	*P* value
Nomura et al. (2003)^34^, ^a^	Japan	28 608	296	Very low vs high	N(−) patients: 5‐y: 76% vs 84% (HR, 1.5; 95% CI, 1.2‐1.9) N(+) patients: 5 y: 24% vs 43% (HR, 1.7; 95% CI, 1.4‐1.9)	<.05
Birkmeyer et al. (2007)^35^	USA	3234	407	High (≥16.5) vs low (≤7.2)	5‐y: 32.0% vs 25.6% (HR, 0.82; 95% CI, 0.73‐0.92)	<.001
Xirasagar et al. (2008)^32^	Taiwan	6909	183	Low (≤57 per 3 y) vs very high (≥358 per 3 y)	5 y: 33% vs 43% (HR, 1.1)	NS
Anderson et al. (2011)^22^, ^b^	England	2758	Unknown	High (>30) vs low (≤10)	Unknown (HR, 0.911)	NS
Dikken et al. (2012)^37^	Netherlands	14 221	91	High (≥21) vs very low (≤5)	Unknown (HR, 0.98; 95% CI, 0.86‐1.12)	NS
Yun et al. (2012)^36^	Korea	66 825	>180	Low (<56) vs high (≥56)	Unknown (HR, 1.36; 95% CI, 1.29‐1.44)	<.05
Coupland et al. (2013)^23^, ^b^	England	7786	144	Highest (≥80) vs lowest (<20)	5‐y: 39% vs 31% (HR, 0.82; 95% CI, 0.72‐0.95)	.0011
Dikken et al. (2013)^24^	Netherlands, Sweden, Denmark, England	9010	Unknown	High (≥21) vs low (≤10)	Unknown (HR, 1.01; 95% CI, 0.84‐1.22)	.561
Smith et al. (2014)^38^	Australia	1621	84	Low (≤6) vs high (>6)	5‐y: 36% vs 40% (HR, 1.11; 95% CI, 0.95‐1.31)	.19

a This study investigated the period between 1975 and 1994, but these values are only for the latest term (1990‐1994).

b These studies included esophageal cancer patients.

CI, confidence interval; HR, hazard ratio; N(−), node negative; N(+), node positive; NS, not significant.

However, several studies could not show a positive relationship between hospital volume and long‐term outcomes.^22^, ^24^, ^32^, ^37^, ^38^ One of these negative studies suggested that this is because long‐term outcomes are affected by two different dimensions of expertise: surgical technical skill and diagnostic ability.^32^ Unfortunately, several of these studies lacked data on such important factors, which might have led to the lack of an observed relationship between hospital volume and long‐term outcomes. Therefore, we now review the results of important studies using data in the landmark phase III trials that made an effort to assure the qualities of surgical or oncological skills and diagnostic ability.

### 3.3 Correlative study of randomized phase III trials

Recently, a correlative study of two randomized phase III trials evaluated the inter‐institutional heterogeneity in short‐ and long‐term outcomes after gastrectomy for resectable gastric cancer.^39^ This study used the data of 521 patients from 23 hospitals in the JCOG9501 trial, which evaluated the survival benefit of the addition of para‐aortic node dissection to standard gastrectomy with D2 lymphadenectomy; and 157 patients from 21 hospitals in the JCOG9502 trial, which evaluated the survival benefit of the left thoracoabdominal approach compared to the abdominal transhiatal approach. This study used the mixed‐effects model to adjust for various background factors. In both trials, some variations among participating institutions were observed in the number of dissected lymph nodes, operative time, and volume of blood loss. Higher hospital volume was significantly correlated with a lower rate of postoperative morbidity in JCOG9501 (*P*=.010) but not in JCOG9502 (*P*=.708). There was no correlation between overall survival (OS) and either hospital volume (JCOG9501, *P*=.617; JCOG9502, *P*=.204) or surgeon volume (JCOG9501, *P*=.776; JCOG9502, *P*=.439). The authors hypothesized that this might have been a result of unknown differences in the prognostic factors of the patients, despite the fact that they all fulfilled the same study inclusion criteria, or differences in surgical skills that were not linked to hospital or surgeon volume.

A second correlative study investigated a phase III chemotherapy trial and evaluated the correlation between hospital volume and outcomes after chemotherapy for unresectable or recurrent gastric cancer. This study used the data of 658 patients from 22 hospitals in the JCOG9912 trial, which compared irinotecan plus cisplatin and S‐1 alone with fluorouracil alone.^40^ Interestingly, a large degree of heterogeneity in median OS was observed for the standard chemotherapy (range, 8.3–13.3 months) even after adjusting for various prognostic factors that could affect outcomes. In contrast, the difference in the estimated median progression‐free survival was only 1.0 months. There was no correlation between hospital volume and OS (*P*=.590), whereas greater medical oncology clinical experience was non‐significantly associated with better OS (*P*=.085) after the standard regimen. Therefore, this study indicated that the large degree of inter‐institutional heterogeneity in OS was mainly a result of the difference in survival after first‐line chemotherapy, which, in turn, may have been a result of variations in medical oncology clinical experience.

## 4 CONCLUSION

As with other types of surgery, heterogeneity in post‐gastrectomy mortality according to hospital volume was observed in multiple studies. Hospital and surgeon volumes have also been reported to be significant factors contributing to heterogeneity in postoperative mortality. However, most studies showed no relationship between hospital volume and postoperative morbidity. The relationship between hospital volume and long‐term outcomes after gastrectomy is also controversial. Recent correlative studies of randomized phase III trials for gastric cancer demonstrated that there was no correlation between postoperative OS and either hospital or surgeon volume, or between OS after chemotherapy and hospital volume.

## DISCLOSURE

Conflicts of Interest: Authors declare no conflicts of interest for this article.
